# Integrated Lymphopenia Analysis in Younger and Older Patients With Multiple Sclerosis Treated With Cladribine Tablets

**DOI:** 10.3389/fimmu.2021.763433

**Published:** 2021-12-24

**Authors:** Gavin Giovannoni, Patricia K. Coyle, Patrick Vermersch, Bryan Walker, Julie Aldridge, Axel Nolting, Andrew Galazka, Caroline Lemieux, Thomas P. Leist

**Affiliations:** ^1^ Blizard Institute, Barts and The London School of Medicine and Dentistry, Queen Mary University of London, London, United Kingdom; ^2^ Department of Neurology, Stony Brook University, Stony Brook, NY, United States; ^3^ Univ. Lille, INSERM U1172, Lille Neurosciences and Cognition, CHU Lille, FHU Precise, Lille, France; ^4^ Department of Neurology, Duke University School of Medicine, Durham, NC, United States; ^5^ Research and Development Global Biostatistics, EMD Serono Research & Development Institute, Inc. (an affiliate of Merck KGaA), Billerica, MA, United States; ^6^ Global Patient Safety, Merck Healthcare KGaA, Darmstadt, Germany; ^7^ Global Clinical Development, Ares Trading SA (an affiliate of Merck KGaA), Eysins, Switzerland; ^8^ North American Medical Affairs, EMD Inc. (an affiliate of Merck KGaA), Mississauga, ON, Canada; ^9^ Comprehensive Multiple Sclerosis Center, Jefferson University, Philadelphia, PA, United States

**Keywords:** cladribine tablets, lymphocyte subsets, lymphopenia, multiple sclerosis, age

## Abstract

Cladribine tablets (CladT) preferentially reduce B and T lymphocyte levels. As aging is associated with a decline in immune function, the effect of CladT on lymphocyte levels may differ by age. This *post hoc* analysis combined data from the Phase 3 CLARITY, CLARITY Extension, and ORACLE-MS studies to examine the effect of age (≤50 or >50 years) on lymphopenia following CladT 3.5 mg/kg (CladT3.5; cumulative dose over 2 years) treatment over 96 weeks. Both CladT3.5 and placebo were given over Weeks 1 and 5 (Year 1 treatment) and Weeks 48 and 52 (Year 2 treatment) from the start of the studies. Absolute lymphocyte count (ALC) and levels of lymphocyte subsets were examined in 1564 patients (Age ≤50 [placebo: N=566; CladT3.5: N=813]; Age >50 [placebo: N=75; CladT3.5: N=110]). In both age groups, following CladT3.5 treatment, nadir for ALC occurred at Week 9 (8 weeks following start of Year 1 treatment) and Week 55 (7 weeks following start of Year 2 treatment) of the 96-week period; for CD19+ B lymphocytes, nadir occurred at Week 9 (Year 1) and Week 52 (Year 2). For CD4+ T lymphocytes, nadir occurred at Week 16 (Year 1) in both age groups, and at Weeks 60 and 72 (Year 2) in the Age ≤50 and >50 groups, respectively. Nadir for CD8+ T lymphocytes occurred at Week 16 (Year 1) and Week 72 (Year 2) in the Age ≤50 group and levels remained in the normal range; nadir occurred at Week 9 (Year 1) and Week 96 (Year 2) in the Age >50 group. Lymphocyte recovery began soon after nadir following CladT3.5 treatment and median levels reached normal range by end of the treatment year in both age groups. By Week 96, ~25% of patients treated with CladT3.5 reported ≥1 episode of Grade ≥3 lymphopenia (Gr≥3L). The rate of certain infections was numerically higher in older versus younger patients who experienced Gr≥3L. In conclusion, CladT3.5 had a similar effect on ALC and lymphocyte subsets in both younger and older patient groups.

**Graphical Abstract:**
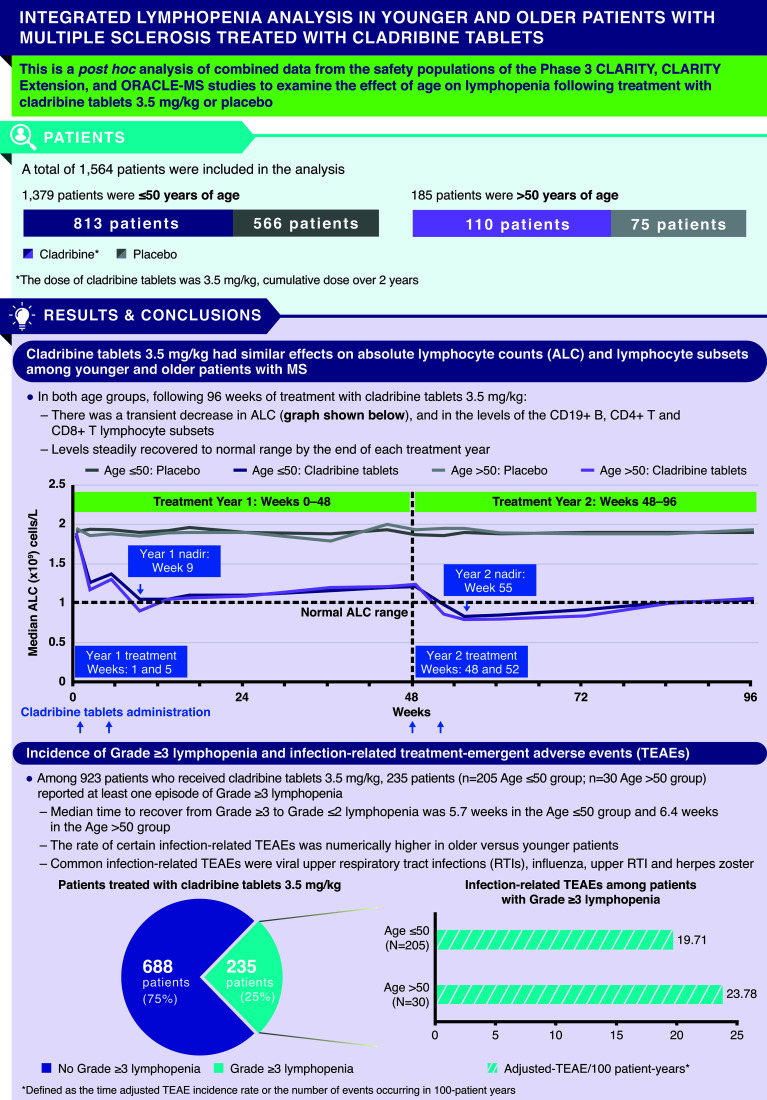


## Introduction

Cladribine tablets are an oral treatment for adults with relapsing forms of multiple sclerosis (MS), which was approved by the European Medicines Agency in 2017 and the US Food and Drug Administration in 2019, and has now received worldwide approval in over 80 countries (1–3). Cladribine treatment is a therapy that induces transient lymphopenia when administered over a very short period, followed by longer treatment-free periods (2, 4, 5). The approved dose of cladribine tablets 10 mg (3.5 mg/kg cumulative dose over 2 years; referred to here as cladribine tablets 3.5 mg/kg) has demonstrated efficacy in patients with relapsing forms of MS, including relapsing-remitting MS (RRMS) (6–10). Cladribine (2-chlorodeoxyadenosine [2-CdA]) is a synthetic, deoxyadenosine analog; it is a prodrug that is preferentially activated in lymphocytes due to their constitutively high deoxycytidine kinase and relatively low 5’-nucleotidase levels (3). Cladribine selectively reduces circulating T and B lymphocytes, which play a central role in the pathogenesis of MS (2, 11). Consistent with the mechanism of action of cladribine, lymphopenia (Grade ≥1) has been reported in ~90% of patients treated with cladribine tablets 3.5 mg/kg over 2 years (7). Analysis of pooled data from the Phase 3 CLARITY and CLARITY Extension trials and the PREMIERE registry showed that treatment with cladribine tablets 3.5 mg/kg resulted in transient decreases in absolute lymphocyte counts (ALC), associated with a reduction in the number of CD19+ B, CD4+ T and CD8+ T lymphocyte subsets (4). This was followed by a recovery phase within weeks of nadir, wherein lymphocyte levels gradually returned to normal levels (4).

The incidence and prevalence of MS have increased over the past decade, with the peak prevalence shifting from the age of 40 years to ~60 years, due to improvements in MS diagnosis and life-expectancy (12, 13). Biological aging of the immune system, known as immunosenescence, is influenced by both genetic and environmental factors and is associated with a reduced ability to fight infections and develop immunological memory (14–17). As immunosenescence occurs with age, total lymphocyte levels are generally lower in older people compared to younger people, therefore use of disease-modifying therapies (DMTs) that further reduce lymphocyte function could potentially put older patients with MS at greater risk of adverse events (12, 15, 18). In addition, age is an important modifier of DMT efficacy; for some DMTs, slowing of MS disability progression decreases with increasing patient age (19). Opportunistic infections such as cryptococcal meningitis are common with some DMTs and this risk increases with age (12). The risk of varicella zoster virus (VZV) reactivation is higher among the elderly (20), and use of DMTs may further increase the risk of viral reactivation among older patients with MS. Low lymphocyte levels, especially in the central nervous system (CNS), are also associated with an increased risk for progressive multifocal leukoencephalopathy (PML) and aging appears to contribute to this risk (18). Given these observations, it is important to understand the immunological impact of DMTs on older versus younger patients with MS.

This *post hoc* analysis aimed to further explore and characterize the impact of age (≤50 years *vs.* >50 years) on the nature of lymphopenia experienced by patients treated with cladribine tablets 3.5 mg/kg in an integrated safety analysis.

## Methods

### Trial Design

CLARITY (NCT00213135) and ORACLE-MS (NCT00725985) were Phase 3, double-blind, randomized, placebo-controlled, 96-week studies of cladribine tablets in patients with RRMS and a first clinical demyelination event, respectively (7, 21). Details have been published previously, but to briefly summarize, both studies evaluated the efficacy and safety of cladribine tablets 3.5 or 5.25 mg/kg (cumulative dose over 2 years) versus placebo in previously treated or untreated patients (7, 21). For the 3.5 mg/kg dose regimen, cladribine tablets (10 mg tablets) were administered over 2 weeks at 0.875 mg/kg/week for 4–5 consecutive days starting on Day 1 of Weeks 1 and 5 of Year 1; this was followed by two further treatment weeks in Year 2 (at Weeks 48 and 52 from the start of study). CLARITY Extension (NCT00641537) was a double-blind, 120-week study that investigated long-term safety and efficacy of cladribine tablets 3.5 mg/kg versus placebo in eligible patients who completed CLARITY. Patients who received placebo in CLARITY were assigned to cladribine tablets 3.5 mg/kg, while those treated with cladribine tablets were re-randomized (2:1) to an additional 2-year course of cladribine tablets 3.5 mg/kg or placebo (9).

### *Post Hoc* Analysis

This *post hoc* analysis was conducted to evaluate the effect of age (Age ≥18–≤50 years [Age ≤50 group] and Age >50–≤65 years [Age >50 group]) at baseline on ALC and levels of lymphocyte subsets (CD19+ B, CD4+ T and CD8+ T lymphocytes) among patients treated with placebo or cladribine tablets 3.5 mg/kg. The analysis period was between Weeks 0–96 of treatment with cladribine tablets 3.5 mg/kg in ORACLE-MS, CLARITY, and CLARITY Extension (treatment Year 1: Weeks 0–48; treatment Year 2: Weeks 48–96). Of the CLARITY patients who entered CLARITY Extension, only patients who received placebo in CLARITY and cladribine tablets 3.5 mg/kg in CLARITY Extension and those who received cladribine tablets 3.5 mg/kg in CLARITY and placebo in CLARITY Extension were included in this analysis. The analysis population was the cladribine tablets 3.5 mg/kg monotherapy oral cohort (4, 7, 9, 21). Assessments included incidence of Grade ≥3 lymphopenia (Gr≥3L); Gr≥3L was defined as ALC levels <500 cells/μL based on the National Cancer Institute Common Terminology Criteria for Adverse Events (NCI CTCAE) Version 3.0 toxicity grading system. The time to recovery from first Gr≥3L to Grade ≤2 lymphopenia (ALC ≥500 cells/μL) was also assessed. All assessments were performed for each age group (≤50 years and >50 years). Among patients who had an episode of Gr≥3L, the rate and type of treatment-emergent adverse events (TEAEs) of viral and bacterial infections was evaluated. The incidence of TEAEs were summarized by Preferred Term (PT) and severity, coded according to the MedDRA dictionary Version 20.0 and expressed in adjusted-TEAE/100 patient-years (Adj-TEAE/100PY). Adj-TEAE/100PY is the time-adjusted incidence rate of TEAEs which can be interpreted as the number of events occurring in 100PY; the confidence interval (CI) is computed with the Wald method for the number of patients with events using a Poisson regression model with fixed effect for treatment group and with log of time at risk as an offset. When the observed rate is zero, the lower limit is set to zero and the exact formula is used for the upper limit. All analyses were performed using Statistical Analysis Software (SAS^®^), Version 9.4 or higher.

## Results

### Patients

A total of 1564 patients were included in this *post hoc* analysis. Of these, 1379 were ≥18 to ≤50 years (Age ≤50 group [placebo: N=566; cladribine tablets 3.5 mg/kg: N=813]) and 185 were >50 to ≤65 years (Age >50 group [placebo: N=75; cladribine tablets 3.5 mg/kg: N=110]) of age. The mean age in the younger age group was 35.1–35.2 years and in the older age group was 53.7–54.0 years (**Table 1**). Baseline demographics and disease characteristics were generally well balanced between age groups. Most patients (79.5–80.9%) were treatment naïve at baseline in both age groups. The two age groups also had broadly similar baseline median ALC (1.86–1.95 x 10^9^ cells/L; **Table 2**) and lymphocyte subset levels (CD19+ B cells: 204–225 cells/µL; CD4+ T cells: 758–952 cells/µL; CD8+ T cells: 338–409 cell/µL; **Supplementary Tables 1**–**3**). Compared with the Age ≤50 group (both placebo- and cladribine tablets-treated patients), the Age >50 group had a higher proportion of women (73.6–76.0% *vs.* 64.8–65.3%), longer disease duration (median 11.4–12.7 years *vs.* 6.4–6.6 years), and more patients with at least one relapse at baseline or in the 12 months prior to study entry (66.3–90.7% *vs.* 53.0–64.5%; **Table 1**). Furthermore, the older age group had a slightly higher proportion of patients with ≥9 T2 lesions (84.9–94.5% *vs.* 85.8–87.2%), but a lower proportion with ≥1 T1 Gd+ lesions (13.6–16.2% *vs.* 33.3–35.5%) at baseline.

**Table 1:** Baseline demographics and disease characteristics of patients in the ≤50 and >50 years age groups.

	Age ≤50 years		Age >50 years	
	Placebo (N=566)	CladT3.5 (N=813)	Placebo (N=75)	CladT3.5 (N=110)
Age, years, mean (SD)				
Mean (SD)	34.9 (8.0)	34.7 (8.4)	54.3 (3.1)	54.6 (3.7)
Median (range)	35.1 (18–50)	35.2 (18–50)	54.0 (50–64)	53.7 (50–65)
Female, n (%)	367 (64.8)	531 (65.3)	57 (76.0)	81 (73.6)
Disease duration, years, median (range)	6.4 (0.4–31.3)^a^	6.6 (0.3–32.8)^b^	12.7 (0.5–39.5)^c^	11.4 (0.4–42.3)^d^
<3 years, n (%)	90 (24.5)	125 (21.5)	7 (10.3)	8 (7.8)
3‒10 years, n (%)	172 (46.9)	291 (50.0)	22 (32.4)	34 (33.0)
>10 years, n (%)	105 (28.6)	166 (28.5)	39 (57.4)	61 (59.2)
Prior use of DMT				
No DMTs	450 (79.5)	650 (80.0)	60 (80.0)	89 (80.9)
1 DMT	91 (16.1)	128 (15.7)	11 (14.7)	16 (14.5)
≥2 DMTs	25 (4.4)	35 (4.3)	4 (5.3)	5 (4.5)
Number of relapses at baseline (12 months prior to study entry), n (%)				
0	201 (35.5)	382 (47.0)	7 (9.3)	37 (33.6)
1	254 (44.9)	305 (37.5)	52 (69.3)	58 (52.7)
≥2	111 (19.6)	126 (15.5)	16 (21.3)	15 (13.6)
EDSS at baseline, median (range)	2.0 (0–5.5)	2.0 (0–6.5)	3.5 (1.0–5.5)	3.5 (0–6.5)
Number of T1 Gd+ lesions at baseline, mean (SD)	0.9 (2.3)^e^	1.2 (3.4)^f^	0.3 (0.9)^g^	0.3 (1.0)
No lesions, n (%)	377 (66.7)	524 (64.5)	62 (83.8)	95 (86.4)
≥1 lesion, n (%)	188 (33.3)	288 (35.5)	12 (16.2)	15 (13.6)
Number of T2 lesions at baseline, mean (SD)	27.7 (22.0)^e^	30.1 (22.3)^f^	21.9 (14.0)^g^	26.2 (13.3)
<9 T2 lesions, n (%)	80 (14.2)	104 (12.8)	11 (15.1)	6 (5.5)
≥9 T2 lesions, n (%)	485 (85.8)	708 (87.2)	62 (84.9)	104 (94.5)
T2 lesion volume (cm^3^), mean (SD)	10.6 (12.2)^e^	11.9 (14.0)^f^	12.8 (12.2)^g^	16.3 (18.8)

^a^n=367, ^b^n=582, ^c^n=68, ^d^n=103, ^e^n=565, ^f^n=812, ^g^n=74. CladT3.5, cladribine tablets 3.5 mg/kg, cumulative dose over 2 years; DMT, disease-modifying therapy; EDSS, Expanded Disability Status Scale; Gd+, gadolinium enhancing; SD, standard deviation.

**Table 2:** Nadir and ALC recovery in years 1 and 2 by age group.

ALC (x10^9^/L), median (IQR)	Age ≤50 years		Age >50 years	
	Placebo (N=566)	CladT3.5 (N=813)	Placebo (N=75)	CladT3.5 (N=110)
**Baseline**				
n	565	811	75	110
ALC	1.91 (1.57, 2.33)	1.86 (1.52, 2.29)	1.95 (1.50, 2.31)	1.89 (1.53, 2.41)
**Year 1**				
**Week 9^a,c^, n**	535	766	72	107
ALC	1.90 (1.56, 2.29)	1.05 (0.80, 1.30)	1.85 (1.48, 2.27)	0.90 (0.72, 1.29)
**Week 48^b,c^, n**	351	511	44	76
ALC	1.87 (1.54, 2.25)	1.21 (0.95, 1.50)	1.93 (1.51, 2.36)	1.24 (1.0, 1.52)
**Year 2**				
**Week 55^a,c^, n**	251	478	29	60
ALC	1.90 (1.56, 2.33)	0.83 (0.60, 1.08)	1.95 (1.59, 2.50)	0.79 (0.57, 1.0)
**Week 96^b,c^, n**	379	573	60	96
ALC	1.90 (1.54, 2.27)	1.04 (0.80, 1.34)	1.93 (1.46, 2.41)	1.06 (0.85, 1.29)

Lower limit of normal = 1.02 x10^9^/L.

a Nadir for ALC in patients treated with CladT3.5.

b Recovery of ALC in patients treated with CladT3.5.

c Week number represents time from start of the study.

ALC, absolute lymphocyte count; CladT3.5, cladribine tablets 3.5 mg/kg, cumulative dose over 2 years; IQR, interquartile range.

### Changes in ALC During 2 Years of Active Treatment With Cladribine Tablets 3.5 mg/kg

**Figure 1:**
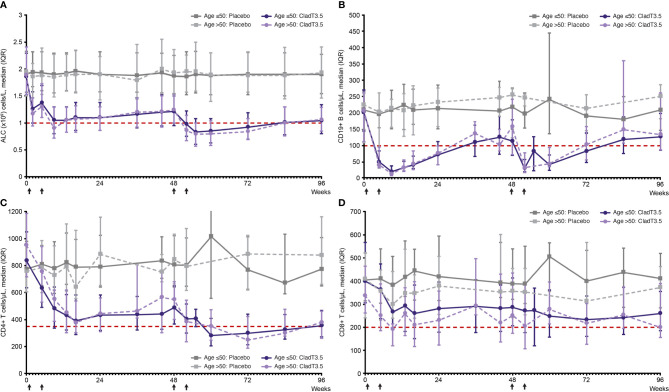
Absolute lymphocyte count and lymphocyte subset levels over time (Week 0–96) from first dose of cladribine tablets 3.5 mg/kg or placebo by age, ≤50 years and >50 years. **(A)** absolute lymphocyte count, **(B)** CD19+ B, **(C)** CD4+ T, and **(D)** CD8+ T lymphocyte subsets. *Notes*: Treatment weeks are indicated by black arrows. Only visits with a sample size ≥10 are shown (On the lymphocyte subset graphs no data plotted for Age ≤50: Placebo [Weeks 36 and 55]; Age >50: Placebo [Weeks 36, 55, 60 and 84]; Age >50: CladT3.5 [Week 55]). Reference line (red) in **(A)** corresponds to ALC lower limit of normal of 1.02 x 10^9^/L. Reference line (red) in **(B–D)** corresponds to lower limit of normal of 100, 350 and of 200 cells/µL, for CD19+ B, CD4+ T and CD8+ T lymphocytes, respectively. ALC, absolute lymphocyte count; CladT3.5, cladribine tablets 3.5 mg/kg; IQR, interquartile range.

In both age groups, the Year 1 (Weeks 0–48) ALC nadir in patients treated with cladribine tablets 3.5 mg/kg occurred at Week 9 and Year 2 (Weeks 48–96) nadir occurred at Week 55 (7 weeks following start of Year 2 dosing; **Figure 1A**). The median (interquartile range [IQR]) ALC at Week 9 for the Age ≤50 and >50 groups was 1.05 (0.80, 1.30) x10^9^ cells/L and 0.9 (0.72, 1.29) x10^9^ cells/L, respectively; median ALC recovered to normal range (above the lower limit of normal [LLN] of 1.02 x10^9^ cells/L) by end of Year 1 (Week 48; **Table 2**). The median (IQR) for the Age ≤50 and >50 groups at Week 55 were below LLN at 0.83 (0.60, 1.08) x10^9^ cells/L and 0.79 (0.57, 1.0) x10^9^ cells/L, respectively. This was followed by a gradual ALC recovery to normal range at the end of Year 2 (Week 96; **Figure 1A**).

### Effects of Cladribine Tablets 3.5 mg/kg on Lymphocyte Subsets

In both study years, a decrease in the levels of CD19+ B, CD4+ T and CD8+ T lymphocytes was observed with cladribine tablets 3.5 mg/kg treatment in both age groups; median lymphocyte levels recovered to normal range by the end of each study year.

#### CD19+ B Lymphocytes

For both age groups, following treatment with cladribine tablets 3.5 mg/kg, the Year 1 nadir for CD19+ B lymphocyte levels occurred at Week 9 and was below LLN; median (IQR) for Age ≤50 and >50 groups were 20 (10, 38) cells/μL and 13 (8, 24) cells/μL, respectively. CD19+ B lymphocyte levels recovered to normal range by Week 36 (**Figure 1B**; **Supplementary Table 1**). Year 2 nadir for CD19+ B lymphocytes occurred at Week 52 (4 weeks following start of Year 2 treatment; median [IQR] for Age ≤50: 31 [20, 58] cells/μL; Age >50: 33 [18, 78] cells/μL; both below LLN), with recovery to normal levels by Week 96.

#### CD4+ T Lymphocytes

Following treatment with cladribine tablets 3.5 mg/kg, Year 1 nadir for CD4+ T lymphocytes occurred at Week 16; median (IQR) for the Age ≤50 and Age >50 groups were 391 (290, 584) cells/µL and 377 (302, 538) cells/µL, respectively. A small increase was observed in CD4+ T lymphocytes after Week 16, and levels remained in the normal range until end of Year 1. In Year 2, nadir for CD4+ T lymphocytes was below LLN in both age groups: median (IQR) was 281 (206, 410) cells/µL for the Age ≤50 group at Week 60 (12 weeks following start of Year 2 treatment) and 250 (189, 423) cells/µL for the Age >50 group at Week 72 (24 weeks following start of Year 2 treatment); levels gradually increased and reached normal range by Week 96 (**Figure 1C**; **Supplementary Table 2**).

#### CD8+ T Lymphocytes

Following treatment with cladribine tablets 3.5 mg/kg, Year 1 nadir for CD8+ T lymphocytes occurred at Week 16 (median [IQR]: 260 [151, 383] cells/µL) in the Age ≤50 group and remained in the normal range. In the Age >50 group, Year 1 nadir occurred at Week 9 (median [IQR]: 191 [120, 215] cells/µL), and recovered to the normal range by end of Year 1. Year 2 nadir occurred at Week 72 (24 weeks following start of Year 2; median [IQR]: 233 [160, 336] cells/µL) in the Age ≤50 group. In the Age >50 group Year 2 nadir occurred later at Week 96 (48 weeks following start of Year 2; median [IQR]: 199 [156, 389] cells/µL). CD8+ T lymphocytes remained in the normal range in Year 2 (**Figure 1D**; **Supplementary Table 3**).

### Grade ≥3 Lymphopenia

#### Incidence of Grade ≥3 Lymphopenia Over 96 Weeks

**Figure 2:**
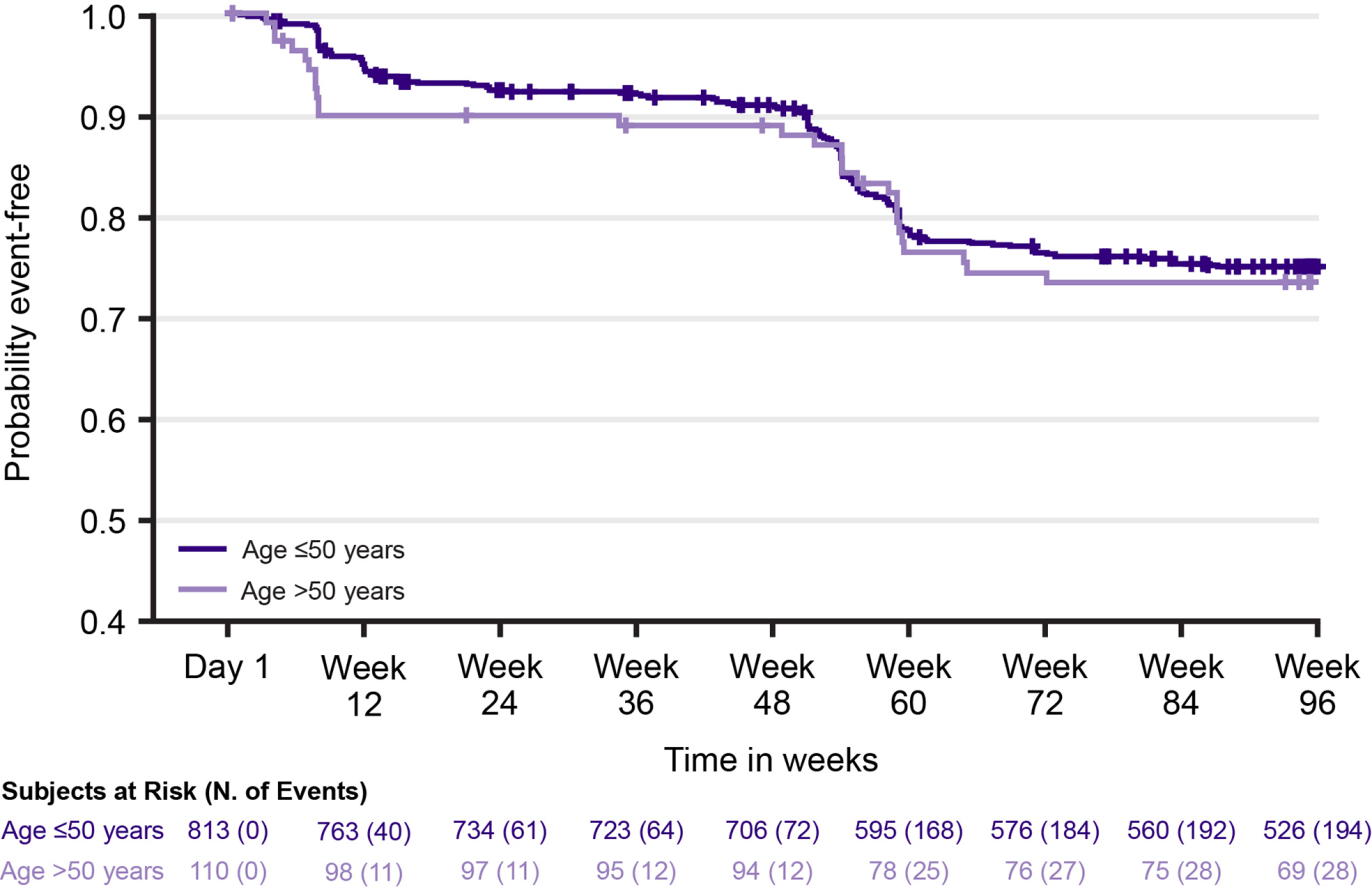
Time to first episode of Grade ≥3 lymphopenia with cladribine tablets 3.5 mg/kg by age group.

In Year 1, following treatment with cladribine tablets 3.5 mg/kg, Gr≥3L was reported in 8.3% and 10.0% of patients in the Age ≤50 and >50 groups, respectively; in Year 2, this increased to 18.7% and 20.0% (**Table 3**). Grade 4 lymphopenia did not occur with cladribine tablets 3.5 mg/kg in Year 1; in Year 2, two (0.3%) patients in the Age ≤50 group and one (1.0%) patient in the Age >50 group experienced Grade 4 lymphopenia. Through Week 96, the overall incidence of Gr≥3L in patients treated with cladribine tablets 3.5 mg/kg was 25.2% (205/813) in the Age ≤50 group and 27.3% (30/110) in the Age >50 group; the mean (standard deviation [SD]) number of Gr≥3L episodes per patient in the respective age groups were 1.6 (1.1) and 1.9 (1.0). **Figure 2** shows the time to onset of first Gr≥3L in the Age ≤50 and >50 groups.

**Table 3:** Incidence of Grade ≥3 lymphopenia in years 1 and 2 by age group.

Patients, n (%)	Age ≤50 years		Age >50 years	
	Placebo (N=566)	CladT3.5 (N=813)	Placebo (N=75)	CladT3.5 (N=110)
Year 1, n	566	808*	75	110
Grade ≥3, n (%)	2 (0.4)	67 (8.3)	0	11 (10.0)
Year 2, n	516	743	68	105
Grade ≥3, n (%)	1 (0.2)	139 (18.7)	0	21 (20.0)

*5 patients did not have post baseline values.

CladT3.5, cladribine tablets 3.5 mg/kg, cumulative dose over 2 years.

#### Time to Recover From Grade ≥3 Lymphopenia

Median (95% CI) time to improvement from Gr≥3L to Grade ≤2 lymphopenia was 5.7 (5.3, 6.1) weeks in the Age ≤50 group and 6.4 (5.3, 8.9) weeks in the Age >50 group. Among patients with Gr≥3L, 87.3% (179/205) in the Age ≤50 group and 80% (24/30) in the Age >50 group remained in the study for 96 weeks. However, whether lymphopenia was the reason for discontinuation in 12.7–20% of patients could not be established given the challenge of retrospectively applying a consistent definition for study discontinuation across different clinical trials.

### Viral and Bacterial Infections in Patients With Grade ≥3 Lymphopenia

The overall incidence of viral and bacterial infections in patients with Gr≥3L following cladribine tablets 3.5 mg/kg treatment was 19.71 (16.28–23.87) and 23.78 (14.78–38.25) Adj-TEAE/100PY (95% CI) for the Age ≤50 and Age >50 groups, respectively (**Table 4**). The most common viral and bacterial infections with cladribine tablets 3.5 mg/kg treatment were (Age ≤50 *vs.* Age >50 in Adj-TEAE/100PY [95% CI]): viral upper respiratory tract infection (RTI; 6.43 [4.84–8.53] *vs.* 4.73 [1.97–11.36]), influenza (3.72 [2.62–5.29] *vs.* 4.83 [2.01–11.60]), upper RTI (3.36 [2.32–4.86] *vs.* 4.60 [1.92–11.06]), and herpes zoster (0.76 [0.36–1.59] *vs.* 3.37 [1.27–8.99]; **Table 4**). In both age groups viral and bacterial infections in patients who received cladribine tablets 3.5 mg/kg were mild to moderate in severity with the exception of one patient in the >50 age group who experienced severe pneumonia and bronchitis (**Supplementary Table 4**).

**Table 4:** TEAEs of viral and bacterial infections in years 1 and 2 among patients who reported Grade ≥3 lymphopenia.

Patients, n (%)	Age ≤50 years				Age >50 years			
	Placebo(N=9)	Adj-TEAE/100PY (95% CI)	CladT3.5(N=205)	Adj-TEAE/100PY (95% CI)	Placebo(N=1)	Adj-TEAE/100PY (95% CI)	CladT3.5(N=30)	Adj-TEAE/100PY(95% CI)
Any viral or bacterial infections	4 (44.4)	9.67 (3.63–25.76)	105 (51.2)	19.71 (16.28–23.87)	0	0 (0–46.95)	17 (56.7)	23.78 (14.78–38.25)
Viral upper RTI	0	0 (0–6.16)	48 (23.4)	6.43 (4.84–8.53)	0	0 (0–46.95)	5 (16.7)	4.73 (1.97–11.36)
Influenza	1 (11.1)	1.93 (0.27–13.67)	31 (15.1)	3.72 (2.62–5.29)	0	0 (0–46.95)	5 (16.7)	4.83 (2.01–11.60)
Upper RTI	2 (22.2)	3.92 (0.98–15.68)	28 (13.7)	3.36 (2.32–4.86)	0	0 (0–46.95)	5 (16.7)	4.60 (1.92–11.06)
Herpes zoster	0	0 (0–6.16)	7 (3.4)	0.76 (0.36–1.59)	0	0 (0–46.95)	4 (13.3)	3.37 (1.27–8.99)

Only TEAEs with >3 Adj-TEAE/100PY are shown.

Adj-TEAE/100PY, Adjusted-TEAE/100 patient-years; CI, confidence interval; CladT3.5, cladribine tablets 3.5 mg/kg, cumulative dose over 2 years; RTI, respiratory tract infection; TEAEs, treatment-emergent adverse events.

## Discussion

The immune system undergoes significant remodeling during aging due to immunosenescence. Studies characterizing the impact and efficacy of DMTs in older patients with MS are limited. Results from this *post hoc* exploratory analysis demonstrated that cladribine tablets 3.5 mg/kg had a similar effect on ALC and lymphocyte subsets in both older and younger patients over 2 years of treatment. In both groups, ALC and levels of lymphocyte subsets decreased in the weeks following cladribine tablets 3.5 mg/kg dosing in both treatment years, and then gradually increased back to normal levels. Additionally, the incidence and duration of Gr≥3L with cladribine tablets 3.5 mg/kg was similar in both younger and older patients with MS.

Decreases in ALC and lymphocyte subsets have been observed in MS patients treated with DMTs such as interferons, dimethyl fumarate (DMF), and alemtuzumab (22). In an integrated analysis of 2470 patients with MS treated with DMF, Gr≥3L lasting ≥6 months was observed in 2.2% of patients (23). Marked reductions of lymphocyte subsets have been observed following infusion of alemtuzumab, an anti-CD52 monoclonal antibody, with a near-complete depletion of ALC, CD19+ B, CD4+ T, and CD8+ T lymphocytes observed (24, 25). Recovery to normal range after alemtuzumab infusion may take from 8 months (B lymphocytes) to nearly 3 years (T lymphocytes) (26). Lymphopenia is an anticipated effect of cladribine tablets due to its mechanism of action; however, recovery of ALC and lymphocyte subsets (CD19+ B and CD4+ T) following reduction due to cladribine tablets starts soon after nadir, reaching normal levels within 36–48 weeks of the start of Year 2 treatment; CD8+ T lymphocytes did not fall below LLN (4). The results from this *post hoc* analysis showed similar trends in both ALC and levels of lymphocyte subsets with recovery to normal ranges occurring by the end of the treatment year in both age groups.

A potential concern associated with lymphocyte depletion for some DMTs, especially in older patients, is an increased risk of opportunistic infections such as PML caused by the John Cunningham virus (JCV). In a multinational cohort of patients with MS, the seroprevalence of JCV increased from 49.5% in patients <30 years to 66.5% in patients over 60 years (27). It has been reported that patients with MS over 50 years of age are at greater risk for developing PML following fingolimod and DMF treatment (18). Older age at the start of natalizumab treatment may lead to a shorter time to onset of PML (28). The additive effects of immunosenescence, as well as natalizumab-induced narrowing of the T cell receptor repertoire and reduction of lymphocyte subsets, have been attributed to this shorter time to PML onset with increasing age (18). No cases of PML have been reported with cladribine tablets to the present date (3). In a prior *post hoc* analysis of patients treated with cladribine tablets 3.5 mg/kg in the monotherapy oral cohort (median age ~36 years), an increased frequency of infections was observed during periods of Gr≥3L; the type of infection events in patients with Gr≥3L did not differ from those occurring outside these episodes (4). This *post hoc* analysis explored the incidence and nature of TEAEs of viral and bacterial infections by age among patients treated with cladribine tablets 3.5 mg/kg who reported Gr≥3L. In this analysis, approximately a quarter of patients treated with cladribine tablets 3.5 mg/kg from each age group experienced at least one episode of transient Gr≥3L. Among patients with Gr≥3L, the rate of certain infection-related TEAEs was numerically higher in older versus younger patients: influenza (4.83 *vs.* 3.72 Adj-TEAE/100PY), upper RTI (4.60 *vs.* 3.36), and herpes zoster (3.37 *vs.* 0.76). The noticeably higher rate of herpes zoster reactivation observed in the Age >50 group is consistent with previous studies, in which viral reactivations were observed in older patients (12, 20).

The current analysis had some limitations. First, it is a *post hoc* analysis of data from previous Phase 3 trials that were not powered to evaluate differences between the younger and older patient groups. Second, most patients (~80%) in this study were treatment naïve prior to study enrollment, which is an unlikely scenario in older patients in the real world. As older patients with MS in the real world are likely to have received prior DMTs, it is unclear how immunosenescence and prior DMT use might impact the effect of cladribine tablets in these patients; this is a subject that requires further research. Recently published real-world studies of cladribine tablets patients (mostly non-elderly) who switched from another DMT showed that cladribine’s effect on lymphocyte changes and clinical and magnetic resonance imaging outcomes were broadly similar in those who were previously untreated compared with those previously treated with DMF, fingolimod, beta-interferons, glatiramer acetate, or teriflunomide (29, 30). Adverse events were also shown to be similar in a small group of patients treated with cladribine, ocrelizumab or rituximab after immediate prior natalizumab use. The effect of cladribine tablets after natalizumab is the subject of ongoing Phase 4 CLADRINA trial (NCT04178005). Third, the Age >50 group was notably smaller than the Age ≤50 group (185 *vs.* 1379); as Adj-TEAE/100PY is based on exposure years, a low sample size will lead to reduced exposure time, which will impact TEAE adjustment. Additionally, TEAE data were not adjusted for duration of a Gr≥3L episode and should be interpreted with caution. Fourth, our analysis of older patients was limited by the fact that the CLARITY study included patients only up to 65 years of age at baseline. In the general population more notable declines in immune response have been reported in people over 65 years of age and these have been associated with a higher vulnerability to infections (31, 32). Fifth, it is important to note that the term ‘immunosenescence’ refers to a quantitative and qualitative decline in immune function with age (33). With aging, there is a marked decline in the production of lymphocytes in the thymus and bone marrow (34). Additionally, qualitative impairments in lymphocytes such as a restricted receptor diversity in T and B cells, and reduced humoral responses against new antigens also occur with aging; these changes contribute to weakened immune response with age (33, 34). Lymphocytes from younger versus older individuals also show distinct gene expression signatures, such as an altered expression of chemokine and cytokine receptors (35). While this current analysis focused on the quantitative differences in lymphocytes among patients treated with cladribine tablets or placebo, qualitative differences in these lymphocytes were not measured. Finally, circulating lymphocytes constitute only ~2% of the total lymphocyte population and, therefore, may not accurately reflect changes that occur within the CNS (22).

## Conclusion

The findings of this *post hoc* analysis of data from Phase 3 studies of cladribine tablets 3.5 mg/kg (CLARITY, CLARITY Extension and ORACLE-MS) demonstrate that cladribine tablets 3.5 mg/kg had a similar effect on ALC, and lymphocyte subsets (CD19+ B, CD4+ T and CD8+ T) in younger and older patients during 2 years of treatment; steady recoveries following nadir were noted in both age groups. The incidence of transient Gr≥3L following treatment with cladribine tablets 3.5 mg/kg was similar between age groups, with ~25% of patients in either group experiencing at least one episode of Gr≥3L. Among patients treated with cladribine tablets 3.5 mg/kg who experienced at least one episode of Gr≥3L, the rate of certain infection-related TEAEs was numerically higher in the older versus younger patient group.

## Data Availability Statement

Any requests for data by qualified scientific and medical researchers for legitimate research purposes will be subject to Merck’s Data Sharing Policy. All requests should be submitted in writing to Merck’s data sharing portal https://www.merckgroup.com/en/research/our-approach-to-research-and-development/healthcare/clinical-trials/commitment-responsible-data-sharing.html. When Merck has a co-research, co-development, or co-marketing or co-promotion agreement, or when the product has been out-licensed, the responsibility for disclosure might be dependent on the agreement between parties. Under these circumstances, Merck will endeavor to gain agreement to share data in response to requests.

## Ethics Statement

CLARITY (NCT00213135), CLARITY Extension (NCT00641537), and ORACLE-MS (NCT00725985) were conducted in accordance with the ethical principles of Declaration of Helsinki and the International Conference on Harmonization (ICH) Harmonized Tripartite Guidelines for Good Clinical Practice (GCP). The study protocols were approved by the institutional review boards and relevant ethics committees of participating centers. The patients/participants provided their written informed consent to participate in this study.

## Author Contributions

Conception, design, and methodology: JA, AN, AG, and CL. Acquisition of data: GG, PKC, PV, BW, and TPL. Analysis of data: JA, AN, AG, and CL. Interpretation of data: GG, PKC, PV, BW, JA, AN, AG, CL, and TPL. Writing, review, and/or revision of the manuscript: GG, PKC, PV, BW, JA, AN, AG, CL, and TPL. All authors contributed to the article and approved the submitted version.

## Funding

This study and manuscript were sponsored by EMD Serono, Inc., Rockland, MA, USA, an affiliate of Merck KGaA (CrossRef Funder ID: 10.13039/100004755).

## Conflict of Interest

The authors declare that this study received funding from EMD Serono, Inc., Rockland, MA, USA, an affiliate of Merck KGaA (CrossRef Funder ID: 10.13039/100004755). The funder had the following involvement with the study: designing the study, collecting and analyzing the data. The authors had full control of the manuscript and provided their final approval of all content. GG has received speaker honoraria and consulting fees from Abbvie, Actelion, Atara Bio, Almirall, Bayer Schering Pharma, Biogen Idec, Celgene-BMS, FivePrime, GlaxoSmithKline, GW Pharma, Janssen, Merck & Co., Merck Healthcare KGaA, Darmstadt, Germany, Pfizer Inc, Protein Discovery Laboratories, Teva Pharmaceutical Industries Ltd, Sanofi-Genzyme, UCB, Vertex Pharmaceuticals, Ironwood, and Novartis; and research support unrelated to this study from Biogen Idec, Merck & Co., Novartis, and Ironwood. PKC declares being an advisor or consultant for Accordant, Biogen, Bristol Myers Squibb, Celgene, Genentech/Roche, Genzyme/Sanofi, GlaxoSmithKline, Janssen, Mylan, Novartis, and Viela Bio, and receiving grants for clinical research from Actelion, Alkermes, Celgene, Corrona LLC, Genentech/Roche, MedDay, NINDS, and Novartis. PV has received honoraria or consulting fees from Biogen, Sanofi-Genzyme, Novartis, Merck Healthcare KGaA, Darmstadt, Germany, Celgene, Roche, AB Science, and Imcyse; and research support from Biogen, Sanofi-Genzyme, and Merck Healthcare KGaA, Darmstadt, Germany. BW has received consulting fees from Biogen, Celgene, EMD Serono, Inc., Rockland, MA, USA, an affiliate of Merck KGaA, Novartis, and Sanofi-Genzyme. JA is an employee of EMD Serono Research & Development Institute, Inc., Billerica, MA, USA, an affiliate of Merck KGaA. AN is an employee of Merck Healthcare KGaA, Darmstadt, Germany. AG was employed by Ares Trading SA, Eysins, Switzerland, an affiliate of Merck KGaA at the time of the study. He is now a consultant for Merck Healthcare KGaA, Darmstadt, Germany. CL is an employee of EMD Inc. Mississauga, Ontario, Canada, an affiliate of Merck KGaA. TPL received consultancy fees or clinical research grants from Acorda, Bayer, Biogen, Daiichi, EMD Serono, Inc., Rockland, MA, USA, an affiliate of Merck KGaA, Darmstadt, Germany, Novartis, ONO, Pfizer and Teva Neuroscience.

## Publisher’s Note

All claims expressed in this article are solely those of the authors and do not necessarily represent those of their affiliated organizations, or those of the publisher, the editors and the reviewers. Any product that may be evaluated in this article, or claim that may be made by its manufacturer, is not guaranteed or endorsed by the publisher.

## Supplementary Material

Supplementary Table 1 Nadir and recovery of CD19+ B lymphocytes in Years 1 and 2 by age group.

Supplementary Table 2 Nadir and recovery of CD4+ T lymphocytes in Years 1 and 2 by age group.

Supplementary Table 3 Nadir and recovery of CD8+ T lymphocytes in Years 1 and 2 by age group.

Supplementary Table 4 Viral and bacterial infections among patients experiencing Gr≥3L, by severity.
