# Supplementary material for: Integrated Lymphopenia Analysis in Younger and Older Patients With Multiple Sclerosis Treated With Cladribine Tablets
Source: Front Immunol. 2021 Dec 24;12:763433. doi: 10.3389/fimmu.2021.763433 (PMC8740297; doi:10.3389/fimmu.2021.763433)
Supplement: Supplementary Table 1 — Nadir and recovery of CD19+ B lymphocytes in Years 1 and 2 by age group. [file DataSheet_1.zip › Supplementary Tables 1-4.pdf]

Giovannoni G, Coyle PK, Vermersch P, Walker B, Aldridge J, Nolting A, Galazka A, Lemieux C and Leist TP (2021) Integrated Lymphopenia Analysis in Younger and Older Patients with Multiple Sclerosis Treated with Cladribine Tablets. *Front. Immunol*. 12:763433. doi: 10.3389/fimmu.2021.763433

SUPPLEMENTARY TABLES

Supplementary Table 1. Nadir and recovery of CD19+ B lymphocytes in Years 1 and 2 by age group

Cells/µL, median (IQR)	Age ≤50		Age >50	
	Placebo (N=566)	CladT3.5 (N=813)	Placebo (N=75)	CladT3.5 (N=110)
**Baseline, n**	90	218	15	32
CD19+ B	210 (149, 268)	204 (145, 271)	225 (109, 259)	208 (113, 264)
**Year 1**				
**Week 9[a,c], n**	65	75	13	13
CD19+ B	209 (152, 269)	20 (10, 38)	214 (156, 242)	13 (8, 24)
**Week 48[b,c], n**	78	137	12	19
CD19+ B	219 (150, 276)	114 (70, 179)	256 (125, 274)	158 (72, 242)
**Year 2**				
**Week 52[a,c], n**	73	165	13	26
CD19+ B	197 (154, 262)	31 (20, 58)	247 (221, 258)	33 (18, 78)
**Week 96[b,c], n**	79	180	13	27
CD19+ B	209 (147, 254)	127 (87, 199)	251 (182, 287)	134 (87, 262)

Lower limit of normal = 100 cells/µL. [a]Nadir for CD19+ B lymphocytes in patients treated with CladT3.5 in that study year; [b]Recovery of lymphocytes in patients treated with CladT3.5; [c]Week number represents time from start of the study.

CladT3.5, cladribine tablets 3.5 mg/kg, cumulative dose over 2 years; IQR, interquartile range.

Giovannoni G, Coyle PK, Vermersch P, Walker B, Aldridge J, Nolting A, Galazka A, Lemieux C and Leist TP (2021) Integrated Lymphopenia Analysis in Younger and Older Patients with Multiple Sclerosis Treated with Cladribine Tablets. *Front. Immunol*. 12:763433. doi: 10.3389/fimmu.2021.763433

Supplementary Table 2. Nadir and recovery of CD4+ T lymphocytes in Years 1 and 2 by age group

	**Age ≤50 years**		**Age >50 years**	
**Cells/µL, median (IQR)**	**Placebo (N=566)**	**CladT3.5 (N=813)**	**Placebo (N=75)**	**CladT3.5 (N=110)**
**Baseline, n**	90	218	15	32
CD4+ T	774 (636, 980)	838 (637, 1050)	758 (605, 1188)	952 (716, 1187)
**Year 1**				
**Week 16[a,c], n**	68	77	12	15
CD4+ T	790 (644, 993)	391 (290, 584)	643 (556, 1063)	377 (302, 538)
**Week 48[b,c], n**	78	137	12	19
CD4+ T	806 (642, 1022)	489 (368, 650)	846 (664, 1033)	549 (402, 681)
**Year 2**				
**Week 60[c], n**	11	109	0	13
CD4+ T	1016 (558, 1381)	281 (206, 410)[a]	0	350 (247, 473)
**Week 72[c], n**	86	186	16	27
CD4+ T	769 (626, 1013)	300 (214, 440)	886 (620, 1025)	250 (189, 423)[a]
**Week 96[b,c], n**	79	180	13	27
CD4+ T	774 (650, 1007)	356 (274, 469)	877 (660, 1162)	380 (261, 463)

Lower limit of normal = 350 cells/µL. [a]Nadir for CD4+ T lymphocytes in patients treated with CladT3.5 in that study year; [b]Recovery of lymphocytes in patients treated with CladT3.5; [c]Week number represents time from start of the study.

CladT3.5, cladribine tablets 3.5 mg/kg, cumulative dose over 2 years; IQR, interquartile range.

Giovannoni G, Coyle PK, Vermersch P, Walker B, Aldridge J, Nolting A, Galazka A, Lemieux C and Leist TP (2021) Integrated Lymphopenia Analysis in Younger and Older Patients with Multiple Sclerosis Treated with Cladribine Tablets. *Front. Immunol*. 12:763433. doi: 10.3389/fimmu.2021.763433

Supplementary Table 3. Nadir and recovery of CD8+ T lymphocytes in Years 1 and 2 by age group

	**Age ≤50 years**		**Age >50 years**	
**Cells/µL, median (IQR)**	**Placebo (N=566)**	**CladT3.5 (N=813)**	**Placebo (N=75)**	**CladT3.5 (N=110)**
**Baseline, n**	90	218	15	32
CD8+ T	405 (314, 522)	400 (306, 567)	409 (240, 523)	338 (262, 464)
**Year 1**				
**Week 9[c], n**	65	75	13	13
CD8+ T	382 (306, 506)	266 (189, 381)	299 (239, 442)	191 (120, 215)[a]
**Week 16[c], n**	68	77	12	15
CD8+ T	445 (318, 538)	260 (151, 383)[a]	347 (240, 426)	210 (116, 398)
**Week 48[b,c], n**	78	137	12	19
CD8+ T	388 (321, 540)	286 (206, 413)	356 (243, 455)	251 (173, 435)
**Year 2**				
**Week 72[c], n**	86	186	16	27
CD8+ T	400 (318, 533)	233 (160, 336)[a]	313 (239, 568)	217 (126, 302)
**Week 96[b,c], n**	79	180	13	27
CD8+ T	411 (285, 520)	260 (185, 355)	372 (283, 462)	199 (156, 389)[a]

Lower limit of normal = 200 cells/µL. [a]Nadir for CD8+ T lymphocytes in patients treated with CladT3.5 in that study year; [b]Recovery of lymphocytes in patients treated with CladT3.5; [c]Week number represents from the start of the study.

CladT3.5, cladribine tablets 3.5 mg/kg, cumulative dose over 2 years; IQR, interquartile range.

Supplementary Table 4. Viral and bacterial infections among patients experiencing Gr≥3L, by severity.

Preferred term, n (%)	Age ≤50 years						Age >50 years					
	Placebo (N=9)			CladT3.5 (N=205)[a]			Placebo (N=1)			CladT3.5 (N=30)		
	**Mild**	**Moderate**	**Severe**	**Mild**	**Moderate**	**Severe**	**Mild**	**Moderate**	**Severe**	**Mild**	**Moderate**	**Severe**
Any TEAEs	4 (44.4)	0	0	46 (22.4)	57 (27.8)	0	0	0	0	4 (13.3)	12 (40.0)	1[b] (3.3)
Viral upper RTI	0	0	0	28 (13.7)	20 (9.8)	0	0	0	0	4 (13.3)	1 (3.3)	0
Influenza	1 (11.1)	0	0	18 (8.8)	13 (6.3)	0	0	0	0	2 (6.7)	3 (10.0)	0
Upper RTI	2 (22.2)	0	0	10 (4.9)	17 (8.3)	0	0	0	0	1 (3.3)	4 (13.3)	0
Herpes zoster	0	0	0	2 (1.0)	3 (1.5)	0	0	0	0	1 (3.3)	3 (10.0)	0

[a]Severity data missing for the following categories: Any TEAE (n=2), Upper RTI (n=1) and Herpes zoster (n=2). [b]One patient reported bronchitis and pneumonia that were severe in intensity. Patients with one or more TEAEs were counted only once on that level. Only TEAEs with >3 adjusted TEAE/100 patient-years are shown.

CladT3.5, cladribine tablets 3.5 mg/kg, cumulative dose over 2 years; Gr≥3L, Grade ≥3 lymphopenia; RTI, respiratory tract infection; TEAE, treatment-emergent adverse event.
